# Effects of Resveratrol on Cognitive Performance, Mood and Cerebrovascular Function in Post-Menopausal Women; A 14-Week Randomised Placebo-Controlled Intervention Trial

**DOI:** 10.3390/nu9010027

**Published:** 2017-01-03

**Authors:** Hamish M. Evans, Peter R. C. Howe, Rachel H. X. Wong

**Affiliations:** School of Biomedical Sciences and Pharmacy, University of Newcastle, Callaghan 2308, New South Wales, Australia; hamish.evans@newcastle.edu.au (H.M.E.); peter.howe@newcastle.edu.au (P.R.C.H.)

**Keywords:** resveratrol, menopause, cerebrovascular function, cognitive decline

## Abstract

We tested whether chronic supplementation with resveratrol (a phytoestrogen) could improve cerebrovascular function, cognition and mood in post-menopausal women. Eighty post-menopausal women aged 45–85 years were randomised to take *trans*-resveratrol or placebo for 14 weeks and the effects on cognitive performance, cerebral blood flow velocity and pulsatility index (a measure of arterial stiffness) in the middle cerebral artery (using transcranial Doppler ultrasound), and cerebrovascular responsiveness (CVR) to both cognitive testing and hypercapnia were assessed. Mood questionnaires were also administered. Compared to placebo, resveratrol elicited 17% increases in CVR to both hypercapnic (*p* = 0.010) and cognitive stimuli (*p* = 0.002). Significant improvements were observed in the performance of cognitive tasks in the domain of verbal memory (*p* = 0.041) and in overall cognitive performance (*p* = 0.020), which correlated with the increase in CVR (*r* = 0.327; *p* = 0.048). Mood tended to improve in multiple measures, although not significantly. These results indicate that regular consumption of a modest dose of resveratrol can enhance both cerebrovascular function and cognition in post-menopausal women, potentially reducing their heightened risk of accelerated cognitive decline and offering a promising therapeutic treatment for menopause-related cognitive decline.

## 1. Introduction

Global dementia data for those over 65 years old indicates that the prevalence of dementia is 14% to 32% higher for women than for men; by the age of 80, women account for 63% of dementia sufferers worldwide and this difference is expected to become more pronounced [1]. This disparity may be at least partially attributable to the loss of estrogen following menopause; declining levels of estrogen have been shown to correlate with deficits in memory, including verbal [2], semantic [3] and visual spatial working memory [4].

Cognitive impairment is associated with reduced cerebral blood flow (CBF) and a reduced ability of cerebral arteries to dilate under dynamic conditions [5,6] and there is evidence to suggest that the loss of estrogen, rather than ageing per se, contributes to reduced cerebrovascular responsiveness (CVR) in post-menopausal women compared with pre-menopausal women and men [7]. Furthermore, women are more likely to suffer from depression following menopause [8], which has been associated with impaired CVR [9]. We have shown that cerebral artery elasticity and adequate cerebral perfusion during mental task activation are predictive of cognitive performance in post-menopausal women and, along with others, that mood deficits can further influence cognitive performance in post-menopausal women [10,11]. Hence, maintaining cerebrovascular function may help optimise mood and attenuate accelerated cognitive decline beyond that of normal ageing in this population [11].

Resveratrol (3,5,4′-trihydroxy-*trans*-stilbene) is a phytoestrogen present in the skin of a range of foods including red grapes, blueberries and peanuts. Resveratrol can act through multiple mechanisms, including binding and activation of estrogen receptors (ER), to increase nitric oxide bioavailability and thereby facilitate the endothelium-dependent vasodilatation necessary for adequate cerebral perfusion [12]. Pre-clinical evidence indicates that resveratrol can improve memory, learning and cognitive reserve, as well as mood, in rats [13,14] and primates [15]. Clinical evidence is however limited. One study demonstrated improvement of verbal memory in older adults [16], while two studies performed in young adults found that administering a single resveratrol dose improved CBF but not cognition [17,18]. We have recently shown that consumption of a single 75 mg dose of resveratrol was most efficacious for enhancing global cerebral vasodilatation [19] and improving performance of a complex cognitive task that required sustained attention in adults with type 2 diabetes, a population with known microvascular dysfunction who also have heightened risk of cognitive impairment [20,21]. The latter was accompanied by enhancement of neurovascular coupling capacity (CVR to the cognitive tasks). We now hypothesise that resveratrol can also enhance cognitive performance and mood by optimising CVR in post-menopausal women.

## 2. Subjects and Methods

### 2.1. Study Design

A 14-week randomised, double-blind, placebo-controlled (parallel comparison) dietary intervention trial evaluated the effects of resveratrol supplementation (75 mg twice daily) on performance to a battery of cognitive tests known to be susceptible to deficits in older women. The trial was conducted at the University of Newcastle in New South Wales, Australia in accordance with the Principles of Good Clinical Practice as outlined by the International Conference on Harmonisation. The protocol was approved by the University of Newcastle’s Human Research Ethics Committee and registered with the Australian and New Zealand Clinical Trial Registry (ANZCTR—ANZCTR12615000291583).

### 2.2. Study Population

To give an 80% chance of detecting a statistically significant difference (*p* < 0.05) of medium effect size (Cohen’s *d* = 0.67) in overall cognitive performance (primary outcome) between resveratrol and placebo treatment, we needed 74 complete data sets; hence, 80 women were recruited to allow for attrition.

Community-dwelling women residing in the Hunter region of New South Wales responded to invitations to participate via an approved media campaign. Respondents completed a health and lifestyle questionnaire to determine their potential suitability. Eligible participants were between 45 and 85 years old, post-menopausal (self-reported cessation of menses for more than six months) and willing to maintain their current lifestyle. Participants were excluded if they were smokers or were taking insulin, warfarin or hormone replacement therapy within the last six months, had suspected dementia, had been diagnosed with depression, had a history of breast or cervical cancer, or had cardiovascular disease, kidney, liver disease or neurological disorders. Written informed consent was obtained prior to any assessments.

### 2.3. Investigational Product and Allocation

The resveratrol and placebo capsules were of identical appearance and supplied by DSM Nutritional Products Ltd. (Kaiseraugst, Switzerland). The resveratrol capsule was comprised of 75 mg of 99% pure synthetic *trans*-resveratrol (ResVida™). The placebo comprised several inert excipients (calcium hydrogen phosphate, microcrystalline cellulose, prosolv 50 and hydrated magnesium silicate). Capsules were identifiable only by code numbers; an independent investigator who held the code allocated participants to treatment groups based on the minimisation method [22]. The first participant was allocated to a group randomly using years since ceasing menses. Blinding was maintained until all data analysis had been completed.

### 2.4. Screening and Baseline Assessments

A detailed description of the outcome assessments has been published [23].

Potentially eligible volunteers attended the screening/baseline visit (week 0), having refrained for at least an hour from medication, food and beverages other than water. Height, weight and waist circumference were measured before seated clinic blood pressure measurements to determine eligibility. Participants then undertook the Australian version of the Modified Mini-Mental State Examination (3MS) to exclude those with suspected dementia (score of <78/100) as outlined by Bravo and Hebert [24].

Transcranial Doppler (TCD) ultrasound (DopplerBox X; Compumedics DWL, Singen, Germany) was used to assess basal cerebral hemodynamics and CVR to both hypercapnic and cognitive stimuli. Eligible volunteers wore a headpiece with bilateral Doppler probes that stayed in place throughout the visit to record mean CBF velocity in the middle cerebral artery (MCA); eighteen participants did not have isolatable TCD signals and underwent cognitive assessment only. The hypercapnic challenge involved inhaling a carbogen gas mixture (5% CO_2_, 95% O_2_) for 180 s. Left and right MCA values were averaged to determine CVR for both hypercapnic and cognitive assessments. CVR to cognitive tests was used as an indirect measure of neurovascular coupling [21]. The cognitive test battery consisted of the Rey Auditory Verbal Learning Test (RAVLT) [25], the Cambridge Semantic Memory Battery [26], the Double Span Task [27] and the Trail Making Task (TMT) [28]. Cognitive tasks were performed in the same order for both the baseline and end-of-intervention visit.

At the conclusion of the cognitive test battery, participants then completed two paper-based questionnaires pertaining to their mood, the Profile of Mood States (POMS) and the Centre for Epidemiologic Studies Depression scale (CES-D) for measures of depressive symptoms.

### 2.5. Intervention

Participants were instructed to take two capsules daily, one in the morning and one in the evening, and record each intake in the assigned supplement diary, in order to aid compliance. Participants were encouraged to maintain their habitual diet throughout the intervention; any changes in dietary supplement and/or medication intake during the intervention were to be recorded in the supplement diary.

A follow-up phone call was made at mid-intervention to enquire about the participants’ well-being and note alterations to their habitual diet, physical activity or medication use. This call also served to encourage compliance. At the end of the trial, all remaining capsules were counted and tallied with the corresponding diary records to calculate overall compliance.

Participants returned at the end of the 14-week intervention, having fasted for at least one hour. They were instructed to refrain from consuming their supplement on the day of their visit. All assessments of basal cerebral hemodynamics, CVR to hypercapnia and cognitive stimuli, cognitive test battery and mood were repeated.

### 2.6. Statistical Analysis

Using an intention-to-treat analysis, treatment-by-time effects were determined by analysis of covariance (ANCOVA) using SPSS version 21.0 (SPSS by IBM Inc., Chicago, IL, USA). The primary outcome was the treatment difference from baseline (week 0) in overall cognitive performance between resveratrol and placebo groups, which was determined by summing the *Z*-scores for each cognitive test. In cognitive tests with more than one component (i.e., RAVLT immediate), *Z*-scores were averaged for each component to form a composite score for that test. *Z*-scores for the final 14-week assessments were derived using the cohort’s mean baseline scores (obtained at week 0). Post-hoc analysis indicated that age, years of education and CES-D scores (depressive symptoms) were all interacting with cognitive performance and, as such, were used as covariates in the analysis, while age also effected CVR and was accounted for accordingly. Effect sizes were calculated using Cohen’s *d*. Secondary outcomes were the treatment-by-time effects on mean CBF velocity, pulsatility index, CVR to hypercapnic provocation, CVR to the cognitive battery and mood states as measured by POMS and CES-D. To determine if the treatment difference in overall cognitive performance was related to changes in CVR to cognitive stimuli, linear correlational analysis was used. To adjust for multiple comparisons in the secondary outcomes, the Bonferroni adjusted level of significance was set at *p* < 0.025.

## 3. Results

### 3.1. Participant Disposition

**Figure 1:**
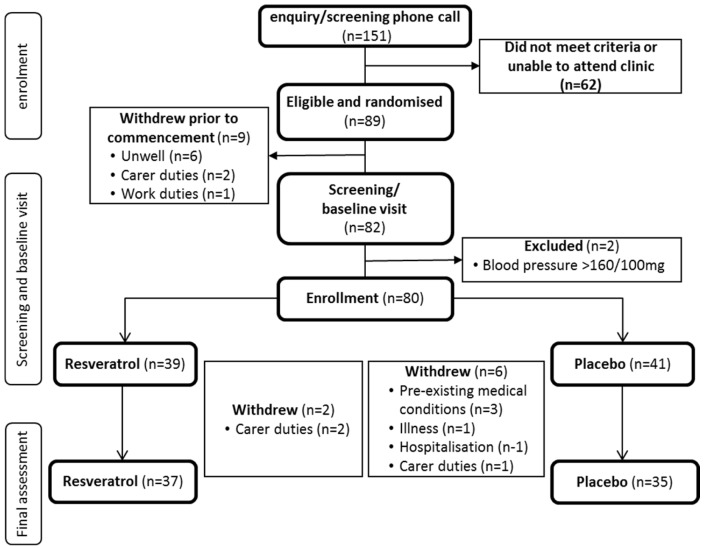
CONSORT diagram. Flow of participants from initial contact until final assessment.

Among the 80 women enrolled, eight women withdrew prior to the end of the intervention. (Figure 1). Three had carer duties and were unable to attend the clinic for their assessments. Another five cited previous medical conditions, sudden illness and hospitalisation unrelated to the study; coincidentally, they had been assigned to the placebo group, indicating resveratrol is safe and tolerable. Due to a significant life event, one participant was identified as a statistical outlier and was excluded from the analysis, as most of her outcome measurements were more than three standard deviations from the mean. Nonetheless, a compliance of 92% was achieved for both groups.

Participant characteristics according to group at baseline are shown in Table 1. They were normotensive, overweight and post-menopausal for approximately 11 years; the range was broad (38 years) but normally distributed. Their high 3MS scores indicated normal cognitive functioning. There were no significant differences in participant characteristics between groups.

### 3.2. Cognitive Performance

**Figure 2:**
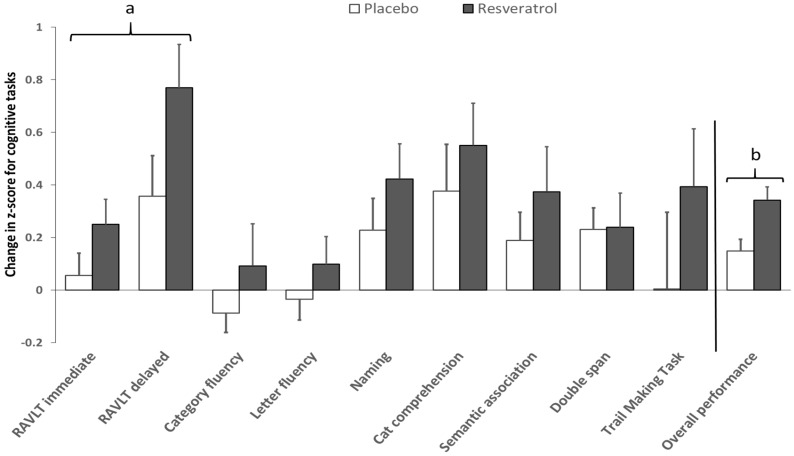
Effects of resveratrol on individual cognitive tests and overcall cognitive performance. Pre-post intervention differences in cognitive performance were calculated for each group using *Z*-scores. Significant differences between resveratrol and placebo; Verbal memory domain ^a^ (*p* = 0.041; Cohen’s *d* = 0.47); overall performance ^b^ (*p* = 0.020; Cohen’s *d* = 0.69) after adjustment for depressive symptoms (Centre for Epidemiologic Studies Depression scale, CES-D), age and years of education. RAVLT = Rey Auditory Verbal Learning Test.

Raw scores (presented as a percentage) and treatment differences for individual cognitive tests and their memory domains are presented in Table 2; when analysing *Z*-scores, the resveratrol group scored better than the placebo group in all individual cognitive tasks (Figure 2). Significant improvements were observed in overall cognitive performance (*p* = 0.003; Cohen’s *d* = 0.69), in addition to the domains of semantic (*p* = 0.043; Cohen’s *d* = 0.48) and verbal memory (*p* = 0.043; Cohen’s *d* = 0.48). However, after controlling for depressive symptoms analysis, only the domain of verbal memory (*p* = 0.037) and overall cognitive performance (*p* = 0.023) remained significantly improved by resveratrol (Figure 2).

### 3.3. Cerebrovascular Function

**Figure 3:**
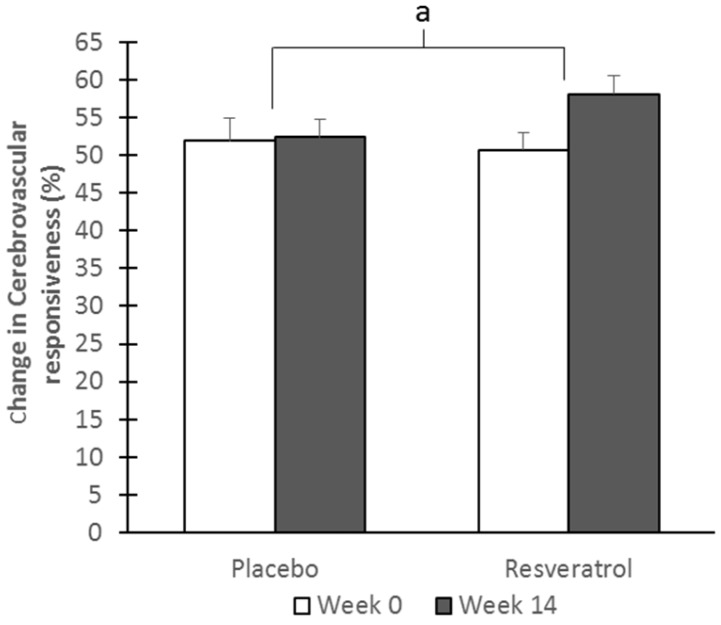
Cerebrovascular responsiveness to hypercapnia in the middle cerebral artery (MCA). Transcranial Doppler (TCD) ultrasound was used to measure blood flow velocity in the MCA in order to calculate treatment changes after 14 weeks in CVR to hypercapnia. ^a^ Significant difference between resveratrol and placebo (*p* = 0.011; Cohen’s *d* = 0.69).

Basal mean CBF velocity (treatment difference; placebo—1.1 ± 1.5, resveratrol—0.2 ± 1.3) and pulsatility index (treatment difference; placebo—0.1 ± 0, resveratrol—0 ± 0) were unaffected by intervention. After adjusting for multiple comparisons, CVR to hypercapnia (Figure 3) was significantly improved, by a magnitude of 7%, with resveratrol compared to placebo (*p* = 0.010; Cohen’s *d* = 0.69) after age adjustments; CVR to the cognitive test battery (Table 3) was also enhanced by resveratrol (*p* = 0.002; Cohen’s *d* = 0.71). This enhancement of CVR by resveratrol was also observed during two individual tasks; the Category fluency and Camel and Cactus (Table 3). Moreover, treatment differences in CVR to the cognitive test battery correlated significantly with the differences in overall cognitive performance (unadjusted *r* = 0.328, *p* = 0.032; adjusted for age, years of education and depressive symptoms *r* = 0.327; *p* = 0.048).

### 3.4. Mood

Following resveratrol supplementation, anxiety (a component of the POMS) was significantly reduced (*p* = 0.025; Cohen’s *d* = 0.50). No significant changes were observed in other components of the POMS or in depressive symptoms, although participants tended to score better in the resveratrol group (Table 4).

## 4. Discussion

This is the first study to demonstrate benefits of chronic resveratrol supplementation (75 mg twice daily for 14 weeks) on cognitive performance and cerebrovascular responsiveness to hypercapnia and cognitive stimuli. Participants were instructed to refrain from consuming their supplement at least 12 h before their final visit; therefore, these improvements likely represent a sustained effect of resveratrol rather than an acute effect, as the half-life of resveratrol in the circulation is approximately nine hours [29]. Additionally, our cohort had high cognitive function, as evident by their 3MS scores and years of education, suggesting that the cognitive benefits elicited by resveratrol may be translated to the general population.

Indeed, overall cognitive performance was significantly better in those taking resveratrol and this trend was reflected in each specific task, though performances did not significantly differ between groups for the latter, which can be attributed to inherent task variability. Given that both the resveratrol and placebo groups improved on their baseline performances, a portion of the improvements may be attributable to a learning effect. Nevertheless, the trend of improvements in overall cognition and domains of verbal and semantic memory in the resveratrol group indicates a potential for resveratrol to improve all memory functions and supports resveratrol supplementation as a potential strategy for attenuating premature cognitive decline in post-menopausal women. We also observed marginal improvements in mood states, indicating the possibility of additional benefits to quality of life in the years following menopause.

This is important clinical evidence, as animal studies of resveratrol supplementation have so far shown improvements in only selective cognitive domains with no overall benefits [13,14,15]. Similarly, in the few human studies, changes in cognition elicited by resveratrol supplementation suggest a benefit for verbal memory alone or no benefit at all. As previously mentioned, Kennedy et al. and Wightman et al. [17,18] found no improvements in cognition with single doses; however, when we consider the acute nature of their study and the young age of their cohorts (~20 years), the lack of effect is not surprising. Our sample population was much older, similar to that studied by Witte et al. [16], who also found improvements following chronic resveratrol treatment but only in verbal memory and not overall cognition. The likely reason our chronic study differs from the findings reported by Witte et al. is that our study was restricted to post-menopausal women, whereas only 40% of their participants were women, of which only nine took resveratrol. Thus, estrogenic actions of resveratrol may provide cognitive benefits additional to those provided through other resveratrol pathways.

We hypothesise that resveratrol elicits its benefits on cognition and also mood through its ability to modulate cerebral perfusion during times of demand. Indeed, allowing for normal aging processes, cognitive decline and, ultimately, dementia are linked to an accelerated decrease in cerebral perfusion [30,31] and impairment of functional hyperaemia within the cerebral cortex [32,33]. With regard to mood, reductions in CBF during major depression mimic those observed in cognitive impairment, although more confined to the frontal regions of the brain [34]. Resveratrol may be influencing CBF through established mechanisms, e.g., Sirtuin 1, AMP-activated protein kinase and Nuclear factor-like 2, to modulate endothelial nitric oxide production [12] and, thereby, cerebral vasodilator function in order to influence mood and cognition. Moreover, resveratrol may also act on cerebral ER to activate endothelial nitric oxide synthase, resulting in increased perfusion in specific brain regions.

While we are unable to demonstrate the direct mechanistic action of resveratrol-induced endothelium-dependent vasodilatation in human cerebral vessels, we did observe that resveratrol significantly improved CVR during both hypercapnic provocation and neuronal activity, suggesting that resveratrol can modulate CBF. This is an important finding when we consider that an absolute 10% reduction in CVR to hypercapnia is associated with a 64% increase in stroke susceptibility [35]. Moreover, we have recently reported that impaired CVR to hypercapnia is associated with poorer cognitive performance in post-menopausal women [11]. Supporting this, others have shown that those with mild cognitive impairment and Alzheimer’s disease have impaired CVR to hypercapnia as measured by Blood Oxygen Level-Dependant spectroscopy [36]. Thus, the ability of resveratrol to improve CVR to hypercapnia is a crucial finding when considered in this context, and given the same trends in cognitive performance, CVR to the cognitive battery and mood, the use of resveratrol to combat accelerated cognitive decline appears promising. Importantly, CVR to the cognitive test battery correlated with overall cognitive performance, indicating that improvments in cognitive performance can be mediated by improvements in neurovascular coupling capacity for post-menopausal women.

Blood samples were not taken in this study, precluding the analysis of biomarkers reflecting other potential mechanisms of action of resveratrol. We acknowledge that the relationship between neurovascular coupling capacity and cognitive performance was merely correlational and additional direct neuroprotective effects of resveratrol in enhancing cognitive performance cannot be ruled out. Evidence indicates that antioxidant effects of resveratrol are able to lessen cerebral ischemic injury [37] and shield neurons from excitotoxicity by counteracting oxidative stress [38]. Additionally, resveratrol, acting through the SIRT1 pathway, can directly suppress the up-regulation of IL-1β and IL-6, inflammatory cytokines released from microglia, which are linked to accelerated memory decline [39,40]. Moreover, in women, resveratrol’s ability to activate cerebral ER may also elicit multifaceted effects on neurons, particularly those located in the hippocampus, increasing the density of dendritic spines and synapses on CA1 pyramidal cells [41] and facilitating NMDA (*N*-methyl-d-aspartate) receptor-mediated responses in these cells [42].

## 5. Conclusions

While the exact mechanisms are still to be confirmed, we have demonstrated that daily resveratrol supplementation for 14 weeks was not only tolerable, but was able to enhance measures of mood and cognitive performance and the latter may be at least partially mediated through improvements in the responsiveness of cerebral vessels to dilate during cognitive demands. These benefits are now being confirmed in a two-year intervention trial in post-menopausal women in which we are also evaluating whether improvements in these clinical outcomes can influence quality of life and everyday functioning (ANZCTR 12616000679482). Findings of these studies are an important step forward in preventative strategies to delay accelerated cognitive decline in our ageing population.

## Figures and Tables

**Table 1:** Participant baseline characteristics (*n* = 79). Data are presented as mean ± SEM (standard error of mean).

Participants’ Characteristics	Placebo (*n* = 41)	Resveratrol (*n* = 38)
Age (years)	61.5 ± 1.2	61.5 ± 1.1
Years since cessation of menses	10.9 ± 1.1	11.8 ± 1.5
Years of formal education	15.4 ± 0.6	15.5 ± 0.7
3MS score (%)	97.8 ± 0.4	98.0 ± 0.3
BMI (Body Mass Index) (kg/m^2^)	26.6 ± 0.8	26.8 ± 0.8
Waist circumference (cm)	87.0 ± 1.4	87.5 ± 1.7
Systolic blood pressure (mmHg)	125.9 ± 2.1	124.7 ± 2.2
Diastolic blood pressure (mmHg)	69.5 ± 1.4	72.4 ± 1.3
Basal mean blood flow velocity (cm/s)	50.3 ± 2.1	48.4 ± 1.8
Pulsatility index	0.8 ± 0.1	0.8 ± 0.1

**Table 2:** Participants’ raw performance data (percentage) for each cognitive component. Data are presented as mean ± SEM.

	Week 0 (V1)		Week 14 (V2)		Δ (V2 − V1)		Placebo vs. Resveratrol	
Memory Domain Component Tasks	Placebo (*n* = 41)	Resveratrol (*n* = 38)	Placebo (*n* = 41)	Resveratrol (*n* = 38)	Placebo (*n* = 41)	Resveratrol (*n* = 38)	*p*-Value (Unadjusted)	*p*-Value (CES-D Covariate)
**Verbal Memory**	50.7 ± 1.2	51.3 ± 1.1	53.2 ± 1.0	56.5 ± 1.1	2.5 ± 0.8	5.2 ± 0.9	0.024 *	0.021 *
RAVLT immediate	52.0 ± 0.8	52.3 ± 0.9	53.9 ± 0.7	55.5 ± 0.8	1.8 ± 0.6	3.2 ± 0.7	0.136	0.135
Learning	66.3 ± 1.7	71.0 ± 2.0	73.6 ± 1.8	78.0 ± 1.9	7.3 ± 1.0	7.0 ± 1.2	0.837	0.830
Proactive memory	46.1 ± 1.2	43.1 ± 1.3	43.8 ± 1.2	44.5 ± 1.5	−2.3 ± 1.6	1.4 ± 1.4	0.160	0.155
Retroactive interference	43.8 ± 1.1	42.7 ± 1.1	44.2 ± 1.2	44.0 ± 1.0	0.4 ± 1.3	1.3 ± 1.4	0.630	0.637
RAVLT delayed	49.3 ± 1.6	50.3 ± 1.6	52.5 ± 1.4	57.5 ± 1.7	3.2 ± 1.2	7.2 ± 1.4	0.035 *	0.029 *
Delayed recall	42.3 ± 1.3	41.4 ± 1.5	43.0 ± 1.2	45.4 ± 1.2	0.7 ± 1.1	3.9 ± 1.8	0.116	0.110
Delayed recognition	56.3 ± 2.3	59.1 ± 2.3	62.1 ± 2.0	69.6 ± 2.6	5.8 ± 2.0	10.5 ± 2.0	0.097	0.073
**Semantic Memory**	75.6 ± 0.7	75.6 ± 0.8	75.9 ± 0.7	76.9 ± 0.8	0.3 ± 0.3	1.3 ± 0.4	0.032 *	0.150
Category fluency	48.7 ± 1.3	48.3 ± 2.0	48.0 ± 1.5	49.2 ± 1.8	−0.7 ± 0.7	0.9 ± 1.0	0.239	0.293
Letter fluency	45.2 ± 2.3	46.4 ± 1.8	45.1 ± 2.0	48.0 ± 2.0	−0.1 ± 1.0	1.6 ± 1.4	0.320	0.348
Naming	97.0 ± 0.3	95.9 ± 0.5	97.6 ± 0.3	97.1 ± 0.4	0.6 ± 0.3	1.2 ± 0.4	0.292	0.290
Category comprehension	99.0 ± 0.2	99.0 ± 0.2	99.4 ± 0.2	99.5 ± 0.1	0.4 ± 0.2	0.6 ± 0.2	0.475	0.587
Camel and cactus	88.0 ± 0.8	87.6 ± 1.1	89.1 ± 0.7	89.9 ± 0.7	1.8 ± 0.6	2.3 ± 1.0	0.353	0.410
**Visuospatial Working Memory**								
Double Span	85.9 ± 0.8	86.0 ± 1.3	87.4 ± 1.0	87.5 ± 1.2	1.5 ± 0.7	1.6 ± 1.1	0.965	0.987
**Executive Function**								
Trail Making Task ^1^								
Time taken A (s)	34.9 ± 1.2	36.0 ± 0.9	33.1 ± 1.2	33.1 ± 1.1	−1.9 ± 1.1	−2.9 ± 1.0	0.512	0.498
Number of Errors A	0.2 ± 0.1	0.1 ± 0.1	0.2 ± 0.1	0.1 ± 0.1	−0.1 ± 0.1	−0.1 ± 0.1	0.973	0.959
Time taken B (s)	72.7 ± 2.9	75.4 ± 3.8	70.5 ± 2.8	68.6 ± 3.1	−2.2 ± 2.9	−6.9 ± 2.6	0.236	0.215
Number of Errors B	0.5 ± 0.1	0.7 ± 0.1	0.5 ± 0.1	0.4 ± 0.1	0.1 ± 0.1	−0.4 ± 0.2	0.043 *	0.084
Interference (ratio)	2.1 ± 0.1	2.1 ± 0.1	2.2 ± 0.1	2.0 ± 0.1	0.1 ± 0.1	−0.1 ± 0.1	0.578	0.602
**Overall Cognitive Performance**	70.6 ± 0.6	70.8 ± 0.7	71.6 ± 0.6	73.1 ± 0.7	1.0 ± 0.3	2.3 ± 0.3	0.004 *	0.018 *

* Independent *t*-test, *p* < 0.05. ^1^ Not presented as a percentage; higher values = worse performance.

**Table 3:** Cerebrovascular responsiveness (percentage) for individual cognitive tasks and overall cognitive cerebrovascular responsiveness (CVR). Data are presented as mean ± SEM.

	Week 0 (V1)		Week 14 (V2)		Δ (V2 − V1)		Placebo vs. Resveratrol	
Outcome Measure	Placebo (*n* = 33)	Resveratrol (*n* = 28)	Placebo (*n* = 33)	Resveratrol (*n* = 28)	Placebo (*n* = 33)	Resveratrol (*n* = 28)	*p*-Value	Adjusted *p*-Value (Age)
RAVLT immediate	16.8 ± 1.6	17.4 ± 2.3	16.4 ± 1.5	21.9 ± 2.2	−0.4 ± 1.6	4.5 ± 1.3	0.020 *	0.033
RAVLT delayed	12.9 ± 1.5	15.2 ± 1.4	13.5 ± 1.3	16.5 ± 1.7	0.6 ± 1.0	1.3 ± 1.2	0.686	0.695
Category fluency	16.1 ± 1.6	6.9 ± 1.9	14.6 ± 0.9	10.1 ± 1.7	−1.4 ± 0.9	3.2 ± 1.7	0.016 *	0.013 *
Letter fluency	9.4 ± 1.9	9.6 ± 1.8	7.4 ± 1.4	19.3 ± 1.7	−1.9 ± 1.9	−0.3 ± 1.7	0.449	0.415
Naming	7.4 ± 1.8	9.7 ± 1.7	8.9 ± 1.4	17.3 ± 1.5	−1.5 ± 1.6	−2.4 ± 1.6	0.097	0.088
Category comprehension	11.4 ± 1.6	9.1 ± 1.5	12.2 ± 1.7	9.3 ± 1.4	0.8 ± 1.6	0.2 ± 1.3	0.761	0.676
Camel and cactus	7.7 ± 1.5	5.1 ± 1.2	4.8 ± 1.0	8.0 ± 1.5	−2.9 ± 1.5	2.8 ± 1.2	0.006 *	0.007 *
Double Span	5.3 ± 1.1	9.5 ± 1.5	6.3 ± 1.3	9.6 ± 1.4	1.0 ± 1.1	0.1 ± 1.4	0.606	0.542
TMT	14.0 ± 2.0	15.8 ± 1.8	11.8 ± 1.9	17.9 ± 2.1	−2.2 ± 1.3	2.1 ± 1.7	0.047	0.046
Overall cognitive CVR	9.6 ± 0.9	10.8 ± 0.8	9.2 ± 0.8	12.6 ± 1.0	−0.4 ± 0.5	1.7 ± 0.5	0.008 *	0.002 *

* Independent *t*-test (Bonferroni adjusted), *p* < 0.025. TMT = Trail Making Task.

**Table 4:** Participants’ mood profiles and individual measures. Data are presented in mean ± SEM.

	Week 0 (V1)		Week 14 (V2)		Δ (V2 − V1)		Placebo vs. Resveratrol
Mood Questionnaire Component	Placebo (*n* = 41)	Resveratrol (*n* = 38)	Placebo (*n* = 41)	Resveratrol (*n* = 38)	Placebo (*n* = 41)	Resveratrol (*n* = 38)	*p*-Value
CES-D ^†^	8.2 ± 1.2	8.5 ± 1.2	9.7 ± 1.4	7.74 ± 1.17	1.5 ± 1.4	−0.8 ± 0.9	0.104
POMS	10.7 ± 3.9	11.5 ± 4.4	8.6 ± 4.8	3.0 ± 4.0	−2.0 ± 2.6	−8.5 ± 2.5	0.085
Anxiety	6.4 ± 0.8	7.4 ± 1.0	6.2 ± 0.8	5.2 ± 0.8	−0.3 ± 0.6	−2.2 ± 0.6	0.025 *
Depression	4.2 ± 0.9	5.6 ± 1.1	4.1 ± 1.2	4.3 ± 0.9	−0.2 ± 0.9	−1.4 ± 0.7	0.290
Anger	5.0 ± 1.0	5.5 ± 1.0	4.2 ± 1.0	4.0 ± 0.9	−0.8 ± 0.7	−1.5 ± 0.7	0.458
Fatigue	6.6 ± 1.0	5.9 ± 0.8	6.7 ± 1.1	4.6 ± 0.8	0.1 ± 0.7	−1.3 ± 9.7	0.159
Confusion	5.4 ± 0.6	5.3 ± 0.6	5.2 ± 0.6	4.5 ± 0.5	−0.2 ± 0.4	−0.7 ± 0.4	0.404
Vigour ^‡^	−17.0 ± 1.0	−18.0 ± 1.0	−17.8 ± 1.1	−19.5 ± 1.0	−0.8 ± 0.7	−1.5 ± 0.9	0.486

^‡^ Vigour scores were reversed. * Independent *t*-test (Bonferroni adjusted), *p* < 0.025. ^†^ A more negative change = improvement in mood with treatment. POMS = Profile of Mood States.

## Abbreviations

CBF	Cerebral blood flow
CVR	Cerebrovascular responsiveness
ER	Estrogen receptors
ANZCTR	Australian and New Zealand Clinical Trial Registry
3MS	Mini Modified Mental State examination
TCD	Transcranial Doppler
MCA	Middle cerebral artery
RAVLT	Rey Auditory Verbal Learning Test
TMT	Trail Making Task
POMS	Profile of Mood States
CES-D	Centre for Epidemiologic Studies Depression scale
ANCOVA	Analysis of covariance
